# Thermal Effects of Pulsed Infrared Lasers on Zirconia Implants at Different Temperatures In Vitro

**DOI:** 10.3390/dj13080342

**Published:** 2025-07-24

**Authors:** George Kokkinos, Maryam Hafeez, Joseph De Leon, Georgios E. Romanos

**Affiliations:** Laboratory for Periodontal-, Implant- and Phototherapy (LA-PIP), Department of Periodontics and Endodontics, School of Dental Medicine, Stony Brook University, Stony Brook, NY 11794, USA

**Keywords:** Er,Cr:YSGG, CO_2_, lasers, peri-implantitis, temperature change

## Abstract

**Objectives:** The aim of this study was to determine the differential temperature produced on ceramic implants using laser irradiation on a pulsed setting of intrabony defects in vitro. **Methods:** A ceramic (Zr) dental implant (Zeramex, 4.8 × 12 mm) was placed into a bovine bone block. A three-wall intrabony defect (6 × 4 × 3 mm) was created to mimic an osseous peri-implant defect. Thermocouples were placed on the apical and coronal areas to measure temperature changes (∆T) during 60 s of laser irradiation. The bovine block was heated to 37 °C, and the defect walls were irradiated with the CO_2_ and Er,Cr:YSGG laser. The settings used were pulsed mode for both lasers, with 30 Hz and 1.5 W for the Er,Cr:YSGG laser and 70 Hz and 2 W for the CO_2_ laser. The same laser settings were repeated at room temperature (RT, 23 °C). Twenty trials were performed for each experimental group at room and body temperature for assessment of ∆T. Paired t-test were used to compare the measurements between 37 °C and 23 °C for the Er,Cr:YSGG, and CO_2_ laser, respectively. **Results:** The CO_2_ laser resulted in the highest ∆T (°C) at the coronal (15.22 ± 0.28/8.82 ± 0.21) and apical (5.84 ± 0.14/2.30 ± 0.28) level when this laser was used in both room temperature and body temperature, respectively. The highest ∆T (°C) for the Er,Cr:YSGG laser at body temperature at the coronal thermocouple was 7.64 ± 0.55, while for the CO_2_ laser, at body temperature was 8.82 ± 0.21. **Conclusion:** Within the limitations of our study, the use of CO_2_ laser and Er,Cr:YSGG laser on peri-implant defects generally appears to be safe in treating peri-implant defects around zirconia implants in vitro.

## 1. Introduction

The incidence of peri-implantitis is a significant problem in the world of dental implants. Peri-implantitis is characterized by supporting alveolar bone loss surrounding the implant body due to microbial colonization and inflammatory destruction [1]. Many strategies for the prevention and treatment of peri-implantitis have been rendered, including scaling and root planning, antibiotics, chemical debridement, regenerative surgery, and implantoplasty [2]. Over the past few years, there has been an increasing popularity for the use of lasers to debride the implant surface. The mechanism of lasers involves the pumping of excited electrons to tissues with high affinity for water, which then causes a rapid thermal expansion of water within the tissues and ablates tissue surfaces [3]. For uses in periodontics, mechanical techniques to scale and debride periodontal tissues can have limitations, including time consumption, difficult root anatomy, and incomplete removal of periodontopathogens [4,5,6,7]. Therefore, dental lasers are advantageous in their ability to exhibit potent bactericidal activity within difficult periodontal pockets and offer efficient removal of calculus and soft matter.

There are a few categories of lasers that are most commonly used in dentistry, and each emits energy at different wavelengths and output settings. The common lasers used in dentistry are diode, carbon dioxide (CO_2_), neodymium-doped yttrium aluminum garnet (Nd: YAG), erbium-doped yttrium aluminum garnet (Er: YAG), and erbium, chromium-doped: yttrium, scandium, gallium, garnet (Er,Cr:YSGG) lasers [8]. The application of lasers in periodontitis and peri-implant therapy stems from the laser’s ability to debride epithelium and connective tissue within periodontal pockets, inhibit bacterial growth, efficiently denude surfaces of calculus, reduce inflammation, and promote cellular proliferation [8,9,10]. Although these lasers have been deemed effective in practice, an adverse effect of using lasers is the overheating of surrounding tissues [11]. It has been reported that the critical temperature of the implant and surrounding tissues is 47 degrees Celsius, where any temperature above that mark for more than 60 s can lead to adverse effects such as thermo-necrosis, bone resorption, and impaired bone regeneration [12,13]. Previous studies have shown that the use of CO_2_ and Er,Cr:YSGG lasers has optimal temperature control in in vitro studies [14,15,16,17] compared to studies on diode lasers [18,19]. CO_2_ lasers emit photonic energy at a wavelength of 9300–10,600 nm [8]. These lasers have the highest absorption in hydroxyapatite and calcium phosphate and are typically used for soft tissue ablation procedures [8]. Typical applications of CO_2_ lasers include debridement of subgingival tissue surrounding implants and bacterial reduction in the gingival sulcus [8,20]. Other lasers, such as the Er,Cr:YSGG laser, which utilizes solid-state crystals incorporated with erbium ions, are useful for ablation of both hard and soft tissue [3]. This is due to the higher absorption coefficient of water (compared to CO_2_) that leads to less tissue degeneration. The wavelength of this laser is 2780 nm and is associated with fewer thermal-related side effects in surrounding tissues [3]. The uses of erbium lasers are very similar to CO_2_ lasers, with the addition of calculus removal [21]. Due to previous studies showing positive applications of CO_2_ and Er,Cr:YSGG, we decided to study the thermal effects of these lasers surrounding zirconia implant surfaces in vitro. However, no protocol exists regarding how to practically ensure that the lasers are kept within this temperature range when treating the periodontal tissues. In this study, we propose practical guidelines for keeping the temperature within the optimum range in peri-implant tissues using CO_2_ and Er,Cr:YSGG lasers in an in vitro study.

## 2. Materials and Methods

A 4.8 × 12 mm ceramic implant (Zeramex; Spreitenbach, Switzerland) was placed into a circular bovine bone block with a diameter of 58 mm and a height of 18 mm (Bonesim, Cassopolis, Michigan, MI, USA). A three-wall intrabony defect of 4 mm width, 4 mm height, and 5 mm depth was created using a high-speed handpiece with a bur. Furthermore, we created a lateral opening near the implant apex in the bone block to allow access for the apical thermocouple. Two thermocouples (T-type Pod; ADInstruments Inc., Colorado Springs, CO, USA) were secured to the coronal and apical surfaces of the implant to monitor the degrees Celsius temperature change using blue periphery wax to secure them in place. The thermocouples were then connected to a data acquisition device (ADInstruments PowerLab 4/35 system and ADInstruments LabChart Software) and computer, which allowed temperature monitoring in real time.

Four experimental groups were created for each laser (CO_2_, Er,Cr:YSGG), and each group consisted of 20 trials; group A, the experimental setting was at room temperature, 23 °C, for 30 s; group B, the experimental setting was at body temperature, 37 °C, for 30 s. Group C, the experimental setting was at room temperature, 23 °C, for 60 s, and group D, the experimental setting was at body temperature, 37 °C, for 60 s. For the CO_2_ laser, there were a total of 80 trials, and for the Er,Cr:YSGG laser, there were a total of 160 trials, as each experimental group was completed twice. The first time, having the water setting of the laser active, and the second time, having the water option inactive. For the body temperature experimental setting, the bovine block was placed inside a 37 °C water bath (Whip Mix, Louisville, KY, USA), and the initial temperature was recorded via the thermocouples. The crest of the bone block was above the water level during the entire duration of the laser irradiation. Irradiation occurred for 30 s and 60 s in each group while the laser handpiece tip was parallel to the long axis of the implant within the intrabony defect. The laser was uniformly moved within the defect in order to equally irradiate all the sites within the defect and around the implant. Two different laser systems, such as a CO_2_ and an Er,Cr:YSGG laser, were utilized (non-contact mode), maintaining a distance of 1–2 mm from the implant surface with pulsed mode settings following the manufacturer guidelines as follows:

1. CO_2_ laser (Denta 2, Lutronic, GPT, Fairfield, NE, USA) with 2 W mean power in a pulsed mode, 700 ms pulse width, and 70 Hz frequency.
2. Er,Cr:YSGG laser (Waterlase MD, Foothill Ranch, CA, USA; Biolase, USA, Irvine, CA, USA) with 1.5 W pulsed mode, 45 mJ, and 30 Hz utilizing MZ5 cylindrical glass tips.

The heat generated during laser irradiation was expressed as ΔT, expressing the change in temperature. The data was recorded and plotted on graphs, and the standard error was computed using a spreadsheet software (Statistical Package, Excel 2023; Microsoft Corporation, Redmond, WA, USA). Statistical analysis with the linear mixed model was used to compare the mean coronal and apical temperature changes in the two different lasers, CO_2_ and Er,Cr:YSGG, at room and body temperature settings.

## 3. Results

### 3.1. Temperature Changes for CO_2_ Laser

In the 23 °C experimental groups (Groups A, C), the highest ΔT was observed for the coronal thermocouple and specifically during the 60 (s) irradiation period (Group C) with a ΔT of 15.22 °C. The coronal thermocouple for the 30 (s) trial followed with a ΔT of 11.28 °C (Group A). Less temperature change was seen at the apical thermocouple. For 60 (s), a ΔT of 5.84 °C was observed (Group C), and the least ΔT was seen for the 30 (s) apical thermocouple (Group A) with a ΔT of 2.65 °C.

**Figure 1:**
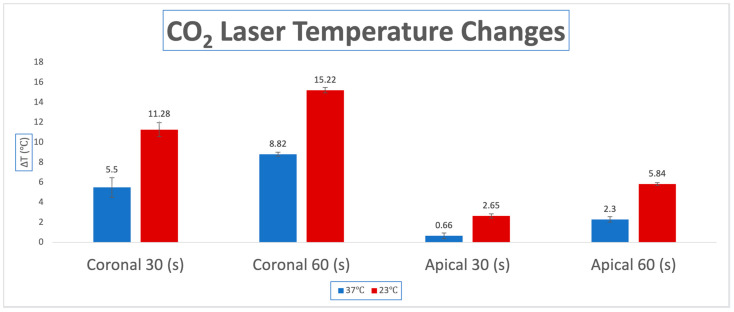
Average temperature changes (ΔT) for apical and coronal thermocouples at 30 and 60 s in room temperature (23 °C) and body temperature (37 °C) for the CO2 laser. Group A: 30 s irradiation at (23 °C); Group B: 30 s irradiation at (37 °C); Group C: 60 s irradiation at (23 °C); Group D: 60 s irradiation at (37 °C).

In the 37 °C body temperature groups (Groups B, D), less ΔT was seen for all the groups comparatively with the 23 °C group. The same pattern was still noted with the coronal 60 (s) group having the largest ΔT of 8.82 °C (Group D), followed by the coronal 30 (s) group with a ΔT of 5.5 °C (Group B). The third largest ΔT was seen for the 60 (s) apical thermocouple, being 2.3 °C (Group D), and the least temperature change was seen for the 30 (s) apical thermocouple, being 0.66 °C (Group B). The results of this experiment can be found in Table 1 and are displayed as a bar graph in Figure 1.

Our statistician performed a paired t-test to compare the measurements at body temperature and room temperature. Both measures at 30 and 60 s show statistically significant differences with a *p*-value of <0.0001 between the two temperature groups.

### 3.2. Temperature Changes for Er,Cr:YSGG Laser

The 23 °C room temperature group without water demonstrated the highest ΔT for both thermocouples (apical and coronal) and irradiation periods (30 and 60 s), which were Group A and Group C. With the 30 (s) coronal ΔT being 13.95 °C, the 60 (s) coronal ΔT being 12.8 °C, the apical 30 (s) 1.24 °C, and the coronal 30 (s) ΔT being 0.41 °C.

The 37 °C body temperature without the water setting showed the second largest change in temperature (ΔT). The largest ΔΤ was observed for the coronal thermocouple at 60 (s) 7.64 °C (Group D), followed by the coronal thermocouple at 30 (s) 7.42 °C (Group B). The apical thermocouple for both 30 and 60 (s) showed a negative ΔT of −0.1 °C for group B and −0.02 °C for group D.

The room temperature 23 °C group with water showed positive temperature change for the coronal 30 and 60 (s) group, ΔT 3.02 °C (Group A) and 2.91 °C (Group C), respectively. Negative changes were seen for the apical 30 (s) thermocouple −0.94 °C (Group A) and apical 60 (s) thermocouple −1.12 °C (Group C).

**Figure 2:**
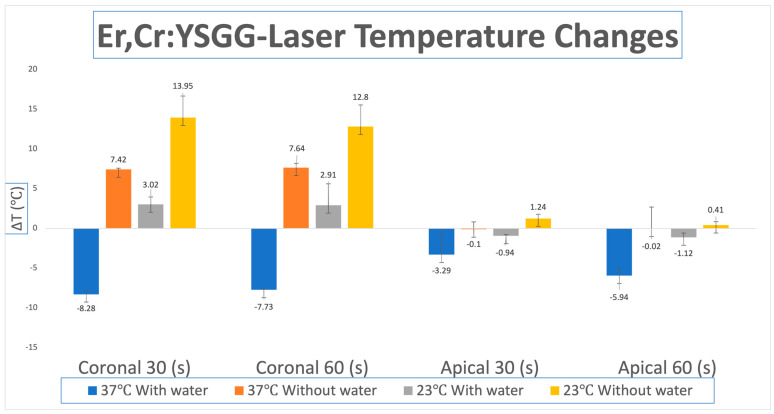
Average temperature changes (ΔT) for apical and coronal thermocouples for 30 and 60 s irradiation in room temperature (23 °C) and body temperature (37 °C) using the Er,Cr:YSGG laser with and without water. Group A: 30 s irradiation at (23 °C); Group B: 30-s irradiation at (37 °C); Group C: 60-s irradiation at (23 °C); Group D: 60-s irradiation at (37 °C).

Lastly, the body temperature (37 °C) with water group showed a ΔT of −8.28 °C for the 30 (s) coronal thermocouple (Group B), −7.73 °C ΔT for the coronal 60 (s) thermocouple (Group D), −3.29 °C ΔT for apical 30 (s) thermocouple (Group B) and a ΔT of −5.94 °C for the apical thermocouple at 60 (s) of irradiation (Group D). Table 2 shows the full results of the experiment and Figure 2 presents them as a bar graph.

Our statistician also performed a paired t-test for the Er,Cr:YSGG laser to compare the measures between body temperature and room temperature when water was present and without water. Both measures at 30 and 60 s show statistically significant difference with a *p*-value of <0.0001 between the two temperature groups with and without water being used.

## 4. Discussion

The literature has shown that the use of Er,Cr:YSGG laser has shown beneficial outcomes in the treatment of chronic periodontitis through the removal of biofilm, endotoxins, infected cementum and calculus, and through a direct stimulatory effect on bone healing [9,10,22,23]. Use of Er,Cr:YSGG lasers has also shown to significantly reduce pocket depths 6 months after treatment [24].

CO_2_ lasers, on the other hand, have shown promising results in their ability to decontaminate the implant surface and significantly reduce the amount of periodontopathogens [11,25]. However, overheating of surrounding tissues has been a significant concern in the use of lasers for periodontal and peri-implant therapy. As the critical threshold for tissue heating is 47 °C, it is of immense priority to ensure that the clinician avoids irradiating the surrounding tissues to this temperature for over 60 s, as succumbing the periodontium to these temperatures can lead to significant adverse effects such as thermal necrosis and bone damage.

A previous study performed by Hafeez et al. [18] tested the thermal effects of diode lasers on titanium implants. The authors used a 3.5 × 11 mm titanium implant and placed it into a round bovine block. Thermocouples were placed on the coronal and apical third of the implant, and two experimental groups were designated at 22 °C and 37 °C. Two experimental groups were designed: Group A (room temperature at 22 °C) and Group B (experimental setting at 37 °C). They irradiated the implant surfaces in each group with different wavelengths of diode, including 810 nm, 940 nm, 970 nm, and 980 nm at pulsed and continuous wave settings. The results of the study showed high temperature changes, well over the critical threshold, at various temperatures and wavelengths of the diode. It is important to note that at 940 nm and 970 nm, the temperature was able to be kept below the critical threshold at body temperature and at a pulsed wave setting [18].

Studies performed by Calce et al. [19] tested the effects of a 445 nm diode laser on titanium and ceramic implants. The study design involved placing a titanium and zirconia implant into an organic dense bone block. Two thermocouple probes were placed on each implant to measure the temperature changes of the implant surfaces in the coronal and apical thirds. Two experimental groups were created: titanium implant at room temperature (21 °C) and in a water bath (37 °C), and zirconia implant at room temperature (21 °C) and in a water bath (37 °C). This study found that in all conditions, apart from zirconia implants at 37 °C, temperature increases above the critical threshold were observed. Titanium implants, in general, were shown to have higher increases in temperature compared to their zirconia counterparts [19]. This is consistent with previous studies that show higher absorption and thermal conductivity in pigmented materials [26,27], but contradicts the findings of Deppe et al. [28] that show greater temperature changes in ceramic compared to titanium.

The above literature provides evidence of diode lasers exceeding the critical threshold at various conditions, which supports our rationale in studying the thermal effects of CO_2_ and Er,Cr:YSGG lasers [11,18,19,25]. The literature seems to suggest that CO_2_ and Er,Cr:YSGG lasers have minimal thermal effects on both hard and soft tissue with appropriate settings and an operator. Therefore, our decision in studying the effects of CO_2_ and Er,Cr:YSGG lasers stems from their thermal superiority and being able to develop a protocol to ensure temperature increases that do not extend beyond the critical threshold. To the best of our knowledge, this is the first study to test the thermal effects of CO_2_ and Er,Cr:YSGG lasers on zirconia implants. The studies mentioned above also justify our decision to study the thermal effects on zirconia (as opposed to ceramic). It seems that the literature indicates a higher temperature increase in titanium as opposed to zirconia, making laser therapy on zirconia implants a safer alternative using the specific laser wavelengths. Future studies should focus on analyzing the thermal effects of CO_2_ and Er,Cr:YSGG lasers on titanium implants to compare the differences between the two materials.

The results from our study show us a few key points. In the analysis of the CO_2_ laser, it is evident that across all trials, a larger temperature change is noted at 60 s compared to 30 s as the implant surface and surrounding bone are being irradiated for a longer period. We can also see that the ΔT in the coronal aspect of the bone–implant interface was higher at both body temperature and room temperature than in the apical portion of the implant. This is reasonable as the coronal portion is in closer contact with the laser irradiation source and will thus be subjected to a greater degree of temperature change compared to the apical portion. In addition, the largest ΔT was observed at room temperature (23 °C) in the coronal portion, which can be explained by the greater initial temperature difference between the irradiation source and the implant compared to body temperature. This temperature increase should be considered when peri-implant defects have to be grafted immediately after implant irradiation.

In the analysis of the Er,Cr:YSGG laser, it was important to differentiate the irradiated surfaces with and without water, as the presence of water promoted a cooling effect that resulted in a decrease in temperature change following radiation. When using the setting with air (without water), we saw an increase in ΔT compared to the groups using water. This setting was used to simulate periodontal defects where the irrigation was unable to reach. Even though the absence of water affected the ΔΤ, the temperature change did not go over the critical threshold. Similarly to the CO_2_ laser, the Er,Cr:YSGG analysis revealed higher temperature changes in the coronal aspect of the implant compared to the apical. However, contrary to the CO_2_ laser analysis, the Er,Cr:YSGG laser analysis revealed no significant differences between irradiated surfaces at 30 s and 60 s, and the highest temperature change was observed at room temperature in the coronal aspect at 30 s irradiation time. Between the two laser wavelengths, we can see that higher temperature changes across the board were noticed in the CO_2_ laser, and the duration of laser exposure to the implant surface also influenced the temperature changes. The differences in temperature changes observed between CO_2_ and Er,Cr:YSGG at 30 s and 60 s can potentially be attributed to the properties of each laser. Because of the high absorption of Er,Cr:YSGG in water [3], lower absorption by the implant surface is expected, which would yield a lower temperature change overall. The CO_2_ lasers in our study have shown higher increases in temperature with increasing duration of exposure, which confirms similar findings in previous studies [14,15,16]. In summary, based on the present in vitro model, the thermal effects of CO_2_ and Er,Cr:YSGG lasers on zirconia implants are kept below the critical temperature threshold.

## 5. Conclusions

As a conclusion, based on the limitations of this study, the present laser irradiation protocol in peri-implant defects seems to be safe for the implant and peri-implant tissues. Further studies will evaluate if this protocol has a positive impact in clinical settings using these specific laser wavelengths.

## Figures and Tables

**Table 1:** CO_2_ temperature changes in degrees Celsius.

CO_2_ Laser Pulsed	Body Temp. 37 °C	Room Temp. 23 °C	*p* Value
	Mean (SD)	Mean (SD)	
**ΔΤ coronal**			
30 s	5.50 (0.98)	11.28 (0.70)	<0.0001
60 s	8.82 (0.21)	15.22 (0.28)	<0.0001
**ΔΤ apical**			
30 s	0.66 (0.28)	2.65 (0.21)	<0.0001
60 s	2.30 (0.28)	5.84 (0.14)	<0.0001

**Table 2:** Er,Cr:YSGG temperature changes in degrees Celsius.

Er,Cr:YSGG-Laser	Body Temp. 37 °C			Room Temp. 23 °C		
	With Water	Without Water	*p*-Value	With Water	Without Water	*p*-Value
**ΔΤ coronal**	**Mean (SD)**			**Mean (SD)**		
30 s	−8.28 (0.44)	7.42 (0.17)	<0.0001	3.02 (2.73)	13.95 (0.92)	<0.0001
60 s	−7.73 (0.17)	7.64 (0.55)	<0.0001	2.91 (0.92)	12.80 (2.70)	<0.0001
**ΔΤ apical**	**Mean (SD)**			**Mean (SD)**		
30 s	−3.29 (0.92)	−0.1 (2.70)	<0.0001	−0.94 (0.17)	1.24 (0.55)	<0.0001
60 s	−5.94 (2.70)	−0.02 (2.73)	<0.0001	−1.12 (0.55)	0.41 (0.44)	<0.0001

## Data Availability

The original contributions presented in the study are included in the article; further inquiries can be directed to the corresponding author.
